# The Combined Extract of Purple Waxy Corn and Ginger Prevents Cataractogenesis and Retinopathy in Streptozotocin-Diabetic Rats

**DOI:** 10.1155/2014/789406

**Published:** 2014-12-31

**Authors:** Paphaphat Thiraphatthanavong, Jintanaporn Wattanathorn, Supaporn Muchimapura, Wipawee Thukham-mee, Kamol Lertrat, Bhalang Suriharn

**Affiliations:** ^1^Department of Physiology and Graduate School (Neuroscience Program), Faculty of Medicine, Khon Kaen University, Khon Kaen 40002, Thailand; ^2^Integrative Complementary Alternative Medicine Research and Development Center, Khon Kaen University, Khon Kaen 40002, Thailand; ^3^Department of Physiology, Faculty of Medicine, Khon Kaen Unieversity, Khon Kaen 40002, Thailand; ^4^Faculty of Agriculture, Khon Kaen University, Khon Kaen 40002, Thailand

## Abstract

Based on the crucial roles of oxidative stress and aldose reductase on diabetic complications and the protective effect against diabetic eye complication of purple waxy corn and ginger (PWCG) together with the synergistic effect concept, we aimed to determine anticataract and antiretinopathy effects of the combined extract of purple waxy corn and ginger (PWCG). The streptozotocin diabetics with the blood glucose levels >250 mg·dL^−1^ were orally given the extract at doses of 50, 100, and 200 mg/kg·BW^−1^ for 10 weeks. Then, lens opacity and histopathology of retina were determined. The changes of MDA together with the activities of SOD, CAT, GPx, and AR in lens were also determined using biochemical assays. All doses of PWCG decreased lens opacity, MDA, and AR in the lens of diabetic rats. The elevation of CAT and GPx activities was also observed. The antiretinopathy property of the combined extract was also confirmed by the increased number of neurons in ganglion cell layer and thickness of total retina and retinal nuclear layer in diabetic rats. PWCG is the potential functional food to protect against diabetic cataract and retinopathy. However, further studies concerning toxicity and clinical trial are still essential.

## 1. Introduction

Diabetic cataract and diabetic retinopathy are chronic progressive, potentially sight-threatening diseases which are associated with hyperglycemia [1, 2]. The epidemiological study has shown that the prevalences of both cataract and retinopathy in diabetic patients are increased [3, 4] accompanied with the increased diabetic patients worldwide. The effective treatment of diabetic cataract and diabetic retinopathy requires the skillful physician. Therefore, these conditions still induce the important health problems in the developing countries especially in a rural area so the prevention strategy is very much important.

Recent findings have demonstrated that the increased aldose reductase activity and the elevated oxidative stress contribute an important role on the development of cataract and retinopathy in diabetes mellitus [5–8]. Anthocyanins rich diet such as purple waxy corn and colored rice could prevent diabetic cataract [9, 10]. It has been reported that purple waxy corn extract decreases oxidative stress in lens of diabetic rats resulting in the decreased cataractogenesis [9]. In addition, ginger extract also exerts anticataractogenesis [11, 12]. Our pilot data show that the antioxidant effect of the combination of purple waxy corn and ginger (PWCG) is more potent than the antioxidant effect of either purple waxy corn or ginger alone (pilot data). However, all of the evidence, mentioned earlier, is the in vitro study. Therefore, we aimed to determine the in vivo effect of PWCG on cataract, retinopathy, oxidative stress, and aldose reductase in lens of diabetic rats.

## 2. Materials and Methods

### 2.1. Plant Material and Extract Preparation

The plant materials used in this study were dried seeds of purple waxy corn or* Zea mays* L. (purple color; KKU open pollinated cultivar) and rhizomes of ginger or* Zingiber officinale* Roscoe. They were harvested during September 2012 and authenticated by Associate Kamol Lertrat and Dr. Bhalang Suriharn, Faculty of Agriculture, Khon Kaen University, Khon Kaen, Thailand. The voucher specimens (voucher specimen 2012001 and 2012002) were kept at the Integrative Complementary Alternative Medicine Research and Development Center, Khon Kaen University. The dried seeds of purple waxy corn and rhizomes of ginger were extracted with 50% hydroalcoholic solvent by maceration method at a ratio of 2 : 5 and 1 : 5 (weight : volume), respectively. The samples were macerated at room temperature for 3 days. Both of the yielded extracts were concentrated by lyophilization and kept at 4°C for further study. The percentage yields of purple waxy corn and ginger extracts were 5.72 and 25.26, respectively. The combination extract was prepared by mixing the purple waxy corn and ginger extracts at a ratio of 1 : 4 (this ratio provided the highest anticataract potential). The contents of total phenolic compounds and anthocyaninsin, the combination extract, were 44.82 ± 2.37 mg/L GAE/mg extract and 392.42 ± 0.03 mg/L cyanidin-3-glucoside equivalents/mg extract, respectively.

### 2.2. Experimental Design

The experimental animals used in this study were male Wistar rats at the weight of 200–250 g (*n* = 8 per group). The animals were maintained and treated in accordance with the guideline and approval of the Ethical Committee on Animals Experiments of Khon Kaen University (AEKKU 98/2555). All rats were divided into various groups as follows.

*Group I*. Control group: all rats in this group were administered citrate buffer, a vehicle of streptozotocin (STZ).

*Group II*. DM + vehicle group: the animals in this group were induced diabetes mellitus via single injection of STZ and received vehicle of the extract or distilled water. 

*Group III–V*. The animals in this group had DM + the combination extract of purple waxy corn and ginger (PWCG) at doses of 50, 100, and 200 mg·kg^−1^ BW, respectively.

All rats in groups II–V were induced diabetes cataract by a single injection of STZ which was dissolved in citrate buffer (pH 4.5) at dose of 55 mg·kg^−1^ BW. The animals which showed the blood sugar levels more than 250 mg·dL^−1^ were recruited for further study. All rats were treated with the assigned interventions once daily at 3 days after injection of STZ and maintained for 10 weeks. Lens opacity was evaluated every week using slit lamp microscope. At the end of study, lens were collected and the level of malondialdehyde (MDA) and the activities of scavenging enzymes activities including superoxide dismutase (SOD), catalase (CAT), and glutathione peroxidase (GPx) in the lens together with lens histomorphology were determined.

### 2.3. Determination of Fasting Blood Glucose Level

After fasting overnight, blood was collected from rat tails and fasting blood glucose levels were determined using ACCU-CHEK active. This process was performed at the first week and every 5 weeks throughout the study period.

### 2.4. Cataract Evaluation via Slit Lamp

Rat eyes were evaluated every week using slit lamp microscope (DIOPTRIX-HAWKEYE; France) by trained observer who was blind to the treatment. The severity of cataract was graded as 5 stages according to the method of Suryanarayana and coworkers [13] as follows.

*Stage 0*. Lens were clear and no vacuoles were observed.

*Stage 1*. Vacuoles cover approximately one-half of the surfaces of the anterior pole forming a subcapsular cataract.

*Stage 2*. Some vacuoles have disappeared and the cortex exhibits a hazy opacity.

*Stage 3*. A hazy cortex remains and dense nuclear opacity is present.

*Stage 4*. A mature cataract is observed as a dense opacity in both cortex and nucleus.

Data were presented as opacity index which was calculated from the following formula:

(1) Opacity  index =number  of  eyes  in  each  stage×stage  of  the  eyetotal  number  of  eyes  within  group.

### 2.5. Homogenate Preparation

At the end of experiment, lens were collected and homogenized in 10% w/v of 0.1 M phosphate buffer, pH 7.4, containing 1 mM EDTA. Then, the homogenate was centrifuged at 10,000 g at 4°C for 1 hour and the supernatant was separated and used for the determination of biochemical parameters.

### 2.6. Determination of Malondialdehyde (MDA) Level

Level of malondialdehyde (MDA), a lipid peroxidation marker, was monitored by using thiobarbituric acid reacting substances (TBARS) assay. In brief, 100 *μ*L of sample was mixed with the solution containing 100 *μ*L of 8.1% (w/v) sodium dodecyl sulphate, 750 *μ*L 20% (v/v) acetic acid (pH 3.5), and 750 *μ*L of 0.8% thiobarbituric acid (TBA). The solution was heated in a water bath at 95°C for one hour and cooled immediately under running tap water. Then, 500 *μ*L chilled water and 2500 *μ*L of butanol and pyridine [15 : 1 v/v] were added into each tube and mixed thoroughly with vortex. Then, the solution was centrifuged at 800 ×g for 20 min. The upper layer was separated and absorbance was measured at 532 nm. 1,3,3-Tetra ethoxy propane (TEP) was used as the reference [14]. The level of MDA was expressed as U/mg protein.

### 2.7. Superoxide Dismutase (SOD) Assay

The determination of SOD activity was carried out via nitro blue tetrazolium (NBT) reduction assay. In this assay, the xanthine-xanthine oxidase system was used as a superoxide generator. In brief, the reaction mixture contained 20 *μ*L of sample and 200 *μ*L of reaction mixture consisting of 57 mM phosphate buffer solution (KH_2_PO_4_), 0.1 mM EDTA, 10 mM cytochrome C solution, and 50 *μ*M of xanthine solution and 20 *μ*L of xanthine oxidase solution (0.90 mU/mL) were prepared at 25°C. The optical density was measured at 415 nm. A system devoid of enzyme served as the control and three parallel experiments were conducted [15]. SOD activity was expressed as U/mg protein.

### 2.8. Catalase (CAT) Assay

Lens catalase activity was determined based on the ability of the enzyme to break down H_2_O_2_. In brief, 10 *μ*L of sample was mixed with the reaction mixture containing 50 *μ*L of 30 mM hydrogen peroxide (in 50 mM phosphate buffer, pH 7.0), 25 *μ*L of H_2_SO_4_, and 150 *μ*L of KMnO_4_. After mixing thoroughly, the optical density was measured at 490 nm. A system devoid of the substrate (hydrogen peroxide) served as the control. The difference in absorbance per unit time was expressed as the activity. The amount of enzyme which is required to decompose 1.0 M of hydrogen peroxide per minute at pH 7.0 and 25° is regarded as one unit [16]. The value of CAT activity was expressed as U/mg protein.

### 2.9. Glutathione Peroxidase (GPx) Assay

This assay was performed based on the glutathione recycling method by using 5,5′-dithiobis (2-nitrobenzoic acid) (DTNB) and glutathione reductase. According to this method, the reaction between DTNB and GSH gave rise to the generation of 2-nitro-5-thiobenzoic acid and GSSG. Since 2-nitro-5-thiobenzoic acid was a yellow colored product, GSH concentration could be determined by measuring absorbance at 412 nm. In brief, a mixture containing a 20 *μ*L of sample and the reaction mixture consisting of 10 *μ*L of dithiothreitol (DTT) in 6.67 mM potassium phosphate buffer (pH 7), 100 *μ*L of sodium azide in 6.67 mM potassium phosphate buffer (pH 7), 10 *μ*L of glutathione solution, and 100 *μ*L of hydrogen peroxide was mixed thoroughly and incubated at room temperature for 5–10 minutes. Then, 10 *μ*L of DTNB (5,5-dithiobis-2-nitrobenzoic acid) was added and the optical density at 412 nm was recorded at 25°C over a period of 5 min. Activities were expressed as nmoles/min/mg lens protein [17]. GPx activity was expressed as U/mg protein.

### 2.10. Aldose Reductase (AR) Activity Assay

Aldose reductase activity was evaluated using spectrophotometric method. An assay mixture containing 0.7 mL of phosphate buffer (0.067 mol), 0.1 mL of NADPH (25 × 10^−5^ mol), 0.1 mL of DL-glyceraldehyde (substrate, 5 × 10^−4^ mol), and 0.1 mL of lens supernatant was prepared. Absorbance was recorded against a reference cuvette containing all other components except the substrate, DL-glyceraldehyde. The final pH of the reaction mixture was adjusted to pH = 6.2. The determination was performed after adding the substrate or DL-glyceraldehyde by measuring the decrease in NADPH absorbance at 390 nm over a 4-minute period [18]. The enzyme activity was expressed as (nmol/min/mg).

### 2.11. Histopathological Analysis of Rat Lens and Retina

The eye balls from five rats per group were fixed in 10% formalin overnight, embedded in paraffin, sectioned at 5 *μ*m thick, and stained with hematoxylin and eosin. Light microscope was used to evaluate the histomorphology of lens. The severity of histomorphological change of lens was graded as a 5-grade score according to the method of Agarwal et al. [19] as described as follow.

*Grade 0*. Presence of anterior epithelium with lens fibers.

*Grade 1*. Presence of anterior epithelium, lens fibers, and vacuoles.

*Grade 2*. Presence of anterior epithelium, lens fibers, vacuoles, and homogenized area. 

*Grade 3*. Absence of anterior epithelium, presence of lens fibers, vacuoles, and homogenized area.

*Grade 4*. Presence of lens fibers and homogenized area only.

Histomorphological changes of retina including the total retinal thickness (from inner limiting membrane to Bruch's membrane), the thickness of the retinal outer nuclear layer, and the number of cells in the ganglion cell layer were also performed. The average thickness of retina was evaluated using 3 adjacent fields and total five images in each group [20]. The results were showed as mean ± SEM.

### 2.12. Statistical Analysis

All data were presented as mean ± standard error mean (mean ± SEM). The analysis of data was performed using one-way analysis of variance (ANOVA) followed by the post hoc test of LSD via SPSS version 15. Statistical differences were considered at *P* value < 0.05.

## 3. Results

### 3.1. Effect of PWCG on Fasting Blood Sugar Level

**Figure 1:**
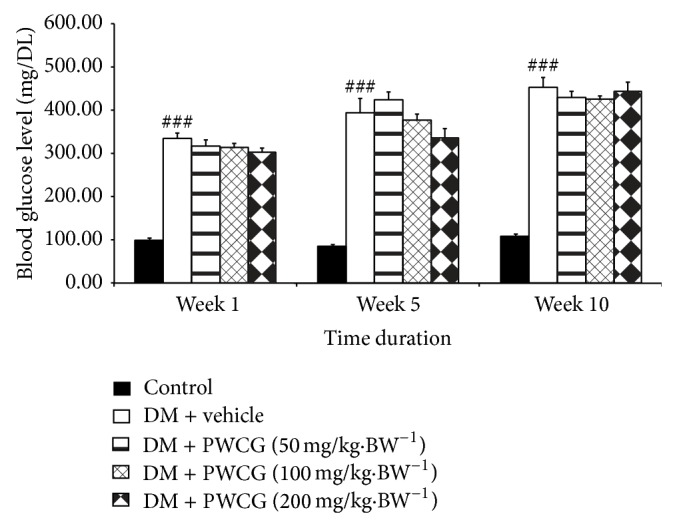
The average fasting blood glucose levels of all the experimental groups at 1-week, 5-week, and 10-week intervention period (*N* = 8/group). ^###^ *P* value < 0.001, compared with control group.

The effect of PWCG on the average fasting blood glucose levels was determined and the results were shown in Figure 1. The results showed that diabetic rats which received distilled water or vehicle used in this study significantly enhanced the blood sugar levels throughout the study period (*P* value < 0.001 all, compared with control rats which received distilled water). It was found that the blood sugar levels of all diabetic rats were more than 300 mg·dL^−1^ throughout the study period. PWCG at all doses used in this study failed to decrease the fasting blood glucose level in diabetic rats.

### 3.2. Anticataract Effect of PWCG

**Figure 2:**
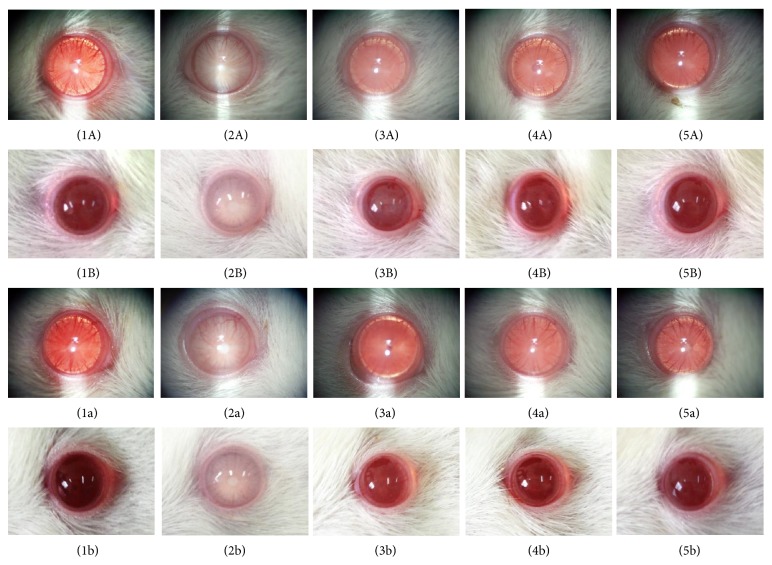
Representative photographs showing the effects of 10-week treatment of PWCG in prevention of cataract. A: photographs via slit lamp, B: photographs via camera; 1: control rat; 2: diabetic rat which received vehicle (DM + vehicle); 3: diabetic rat which received PWCG at dose of 50 mg·kg^−1^ BW (DM + PWCG 50 mg·kg^−1^ BW); 4: diabetic rat which received PWCG at dose of 100 mg·kg^−1^ BW (DM + PWCG 100 mg·kg^−1^ BW); 5: diabetic rat which received PWCG at dose of 200 mg·kg^−1^ BW (DM + PWCG 200 mg·kg^−1^ BW).

The effect of PWCG on lens opacity was shown in Figure 2. The lens opacity in control rats also developed cataract during 6–10-week period. At 10-week study period, all lens from both right (1A and 1B) and left eyes (1a and 1b) of control rats were clear and normal. Both right (2A and 2B) and left eyes (2a and 2b) of diabetic rats which received vehicle showed lens opacity. However, the increased lens opacity induced by diabetic condition was attenuated in diabetic rats which received all doses of PWCG (3A–5A, 3B–5B, 3a–5a, and 3b–5b).

**Figure 3:**
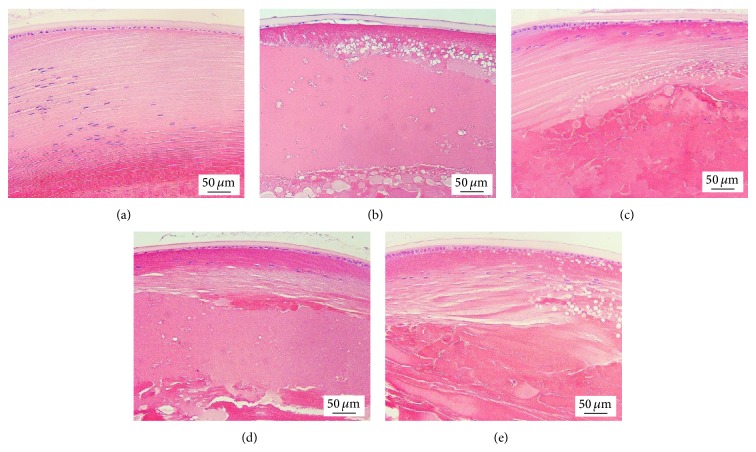
Photographs of transverse sections of eye balls (5 μm thickness), stained with H&E, which determined the cataract severity at the end of experiment using light microscope (week 10). (a) Control, (b) DM + Vehicle, (c) DM + PWCG (50 mg/kg·BW), (d) DM + PWCG (100 mg/kg·BW), and (e) DM + PWCG (200 mg/kg·BW).

Table 1 showed that the lens opacity indices of diabetic rats which received vehicle started to increase at 3-week treatment duration (*P* value < 0.01, compared with control rats). This change was still observed until the end of study (*P* value < 0.001, compared with control rats). Diabetic rats which received PWCG at dose of 200 mg·kg^−1^ significantly mitigated the elevation of lens opacity index induced by diabetes at 5-week treatment (*P* value < 0.05, compared with diabetic rats which received vehicle). This change persisted until the end of study (*P* value < 0.01, 0.05, 0.001, 0.001, 0.001 resp., compared with diabetic rats which received vehicle). Diabetic rats which received PWCG at low dose (50 mg·kg^−1^ BW) also mitigated the elevation of lens opacity index induced by diabetes but the significant changes were observed at 3-week, 5 week, 8-week, 9-week, and 10-week treatment duration (*P* value < 0.05, 0.05, 0.05, 0.001 and 0.001 resp., compared with diabetic rats which received vehicle). However, the medium dose of PWCG produced the significant mitigation effect on lens opacity index induced by diabetes only at 8-week, 9-week, and 10-week treatment duration (*P* value < 0.05, 0.001 and 0.001 resp., compared with diabetic rats which received vehicle). The effects of PWCG on lens opacity were confirmed by histopathological analysis of lens as shown in Figure 3. It was found that, at 10-week study period, the lens capsule thickness and the density of epithelial cells of diabetic rat subjected to vehicle treatment were decreased.

In addition, the reduction of differentiated lens fibers, vacuoles, and homogenized area was also observed (Figure 3(b)). Diabetic rats which received PWCG at doses of 50, 100, and 200 mg·kg^−1^ BW showed the increased density of epithelial cells and differentiated lens fiber thickness together with the decreased vacuoles and homogenized area (Figures 3(c), 3(d), and 3(e)).

**Figure 4:**
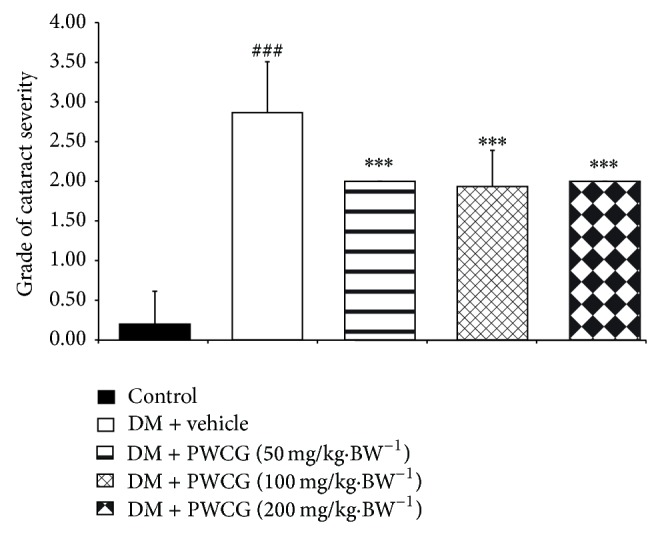
The cataract severity of control and diabetic rats (DM) which received either vehicle or PWCG at doses of 50, 100, and 200 mg·kg^−1^ BW for 10 weeks. ^###^ *P* value < 0.001, compared with control, and ^***^ *P* value < 0.001, compared with vehicle.

Figure 4 showed the effect of PWCG on the severity of lens damage. Our results showed that the diabetic rats which received vehicle showed the increased severity of cataract (*P* value < 0.001 compared with control rats). All doses of PWCG significantly mitigated the enhanced cataract severity induced by diabetes mellitus (*P* value < 0.001 all, compared with diabetic rats which received vehicle).

### 3.3. The Effect of PWCG on Retinopathy in Diabetic Rats

**Figure 5:**
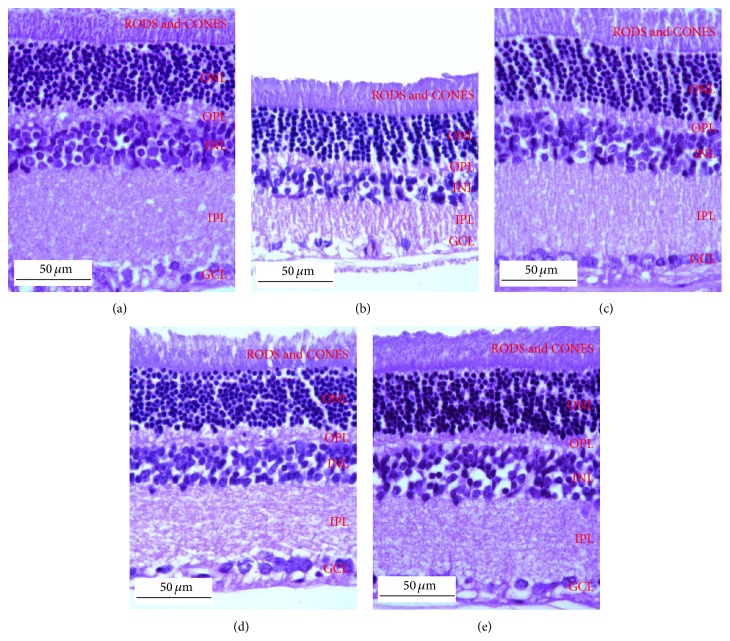
Light microscope of transverse section of eye ball (5 μm thickness), stained with H&E, which determined the pathological change of retina at the end of experiment (week 10). (a) Control rat; (b) diabetic rat which received vehicle (DM + vehicle); (c) diabetic rat which received PWCG at dose of 50 mg·kg^−1^ BW (DM + PWCG 50 mg·kg^−1^ BW); (d) diabetic rat which received PWCG at dose of 100 mg·kg^−1^ BW (DM + PWCG 100 mg·kg^−1^ BW); (e) diabetic rat which received PWCG at dose of 200 mg·kg^−1^ BW (DM + PWCG 200 mg·kg^−1^ BW).

**Figure 6:**
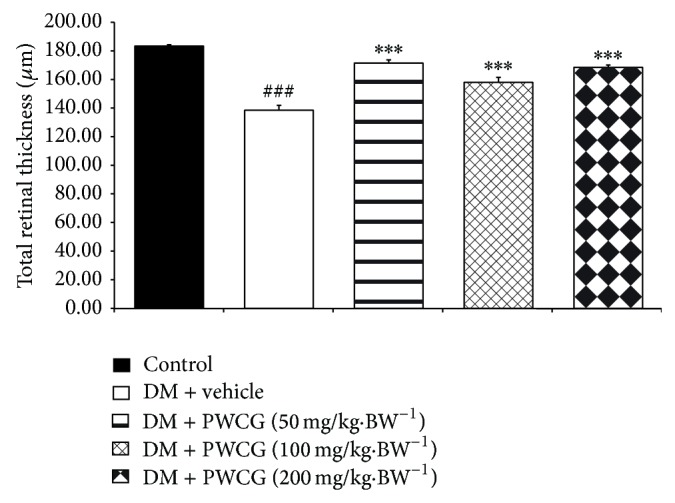
The total retinal thickness of retina (TRT) of control and diabetic rats (DM) which received either vehicle or the combination extract of purple waxy corn and ginger (PWCG) at doses of 50, 100, and 200 mg·kg^−1^ BW for 10 weeks. ^###^ *P* value < 0.001, compared with control, and ^***^ *P* value < 0.001, compared with vehicle.

**Figure 7:**
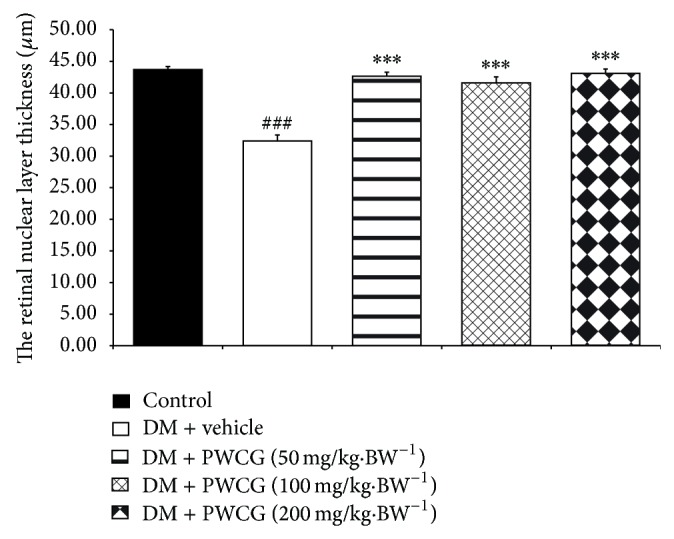
The thickness of the retinal outer nuclear layer (ROT) of control and diabetic rats (DM) which received either vehicle or the combination extract of purple waxy corn and ginger (PWCG) at doses of 50, 100, and 200 mg·kg^−1^ BW for 10 weeks. ^###^ *P* value < 0.001, compared with control, and ^***^ *P* value < 0.001, compared with vehicle.

**Figure 8:**
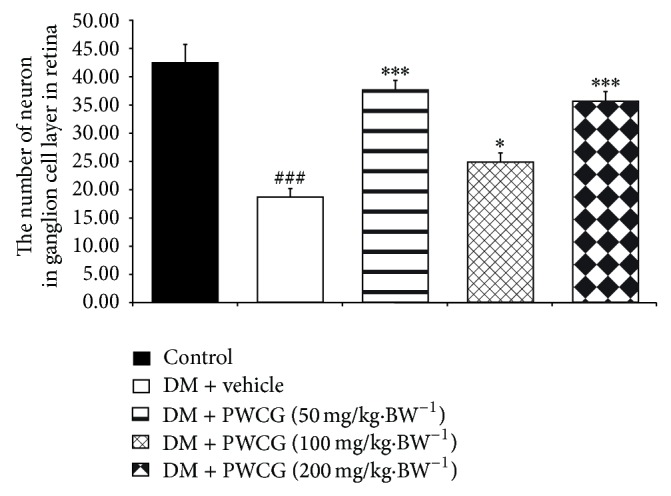
The number of cells in the ganglion cell layer (NG) in retina of control and diabetic rats (DM) which received either vehicle or the combination extract of purple waxy corn and ginger (PWCG) at doses of 50, 100, and 200 mg·kg^−1^ BW for 10 weeks. ^###^ *P* value < 0.001, compared with control, and  ^*^, ^***^ *P* value < 0.05 and 0.001, compared with vehicle.

The effect of PWCG on histopathological change of retina was shown in Figure 5. At 10-week study period, the total retinal thickness (TRT), the thickness of the retinal outer nuclear layer (ROT), and the number of cells in the ganglion cell layer (NG) of diabetic rats which received vehicle significantly decreased when compared to control. However, the decreased TRT, ROT, and NG induced by diabetic condition were attenuated in diabetic rats which received all doses of PWCG. Figures 6, 7, and 8 showed that diabetic rats which received PWCG at doses of 50, 100, and 200 mg·kg^−1^ BW significantly increased the TRT (*P* value < 0.001 all, compared with diabetic rats which received vehicle), ROT (*P* value < 0.001 all, compared with diabetic rats which received vehicle), and NG of retina (*P* value < 0.001, 0.05 and 0.001 resp., compared with diabetic rats which received vehicle).

### 3.4. Effect of PWCG on Oxidative Stress Markers

**Figure 9:**
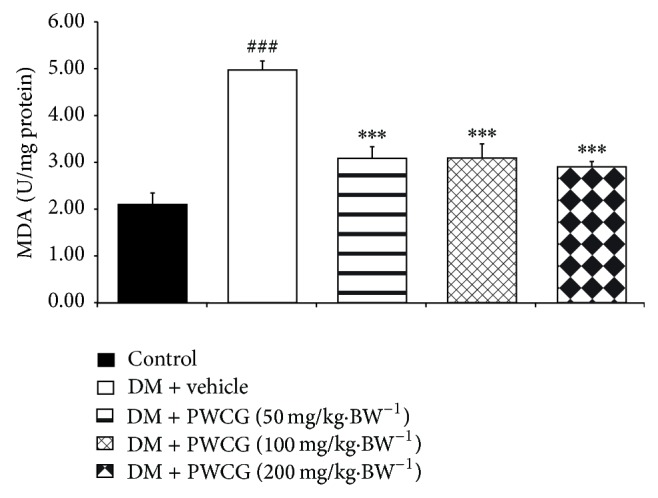
The level of malondialdehyde (MDA) (U/mg protein) in lens of control and diabetic rats (DM) which received either vehicle or the combination extract of purple waxy corn and ginger (PWCG) at doses of 50, 100, and 200 mg·kg^−1^ BW for 10 weeks (*N* = 8/group). ^###^ *P* value < 0.001, compared with control, and ^***^ *P* value < 0.001, compared with diabetic rats which received vehicle (DM + vehicle).

**Figure 10:**
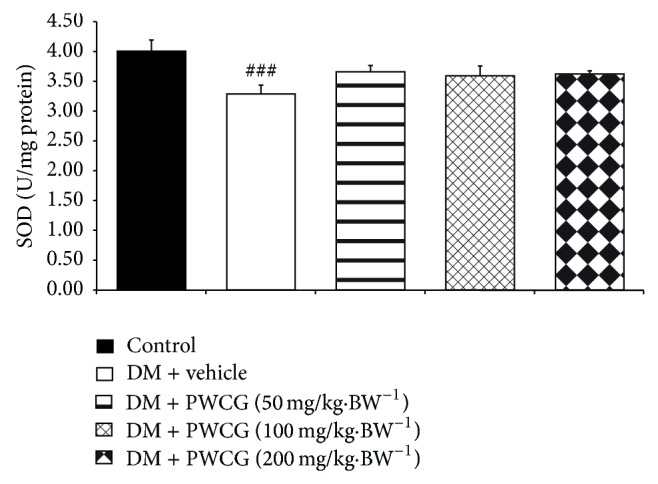
The activity of superoxide dismutase (SOD) (U/mg protein) in lens of control and diabetic rats (DM) which received either vehicle or the combination extract of purple waxy corn and ginger (PWCG) at doses of 50, 100, and 200 mg·kg^−1^ BW for 10 weeks (*N* = 8/group). ^###^ *P* value < 0.001, compared with control.

**Figure 11:**
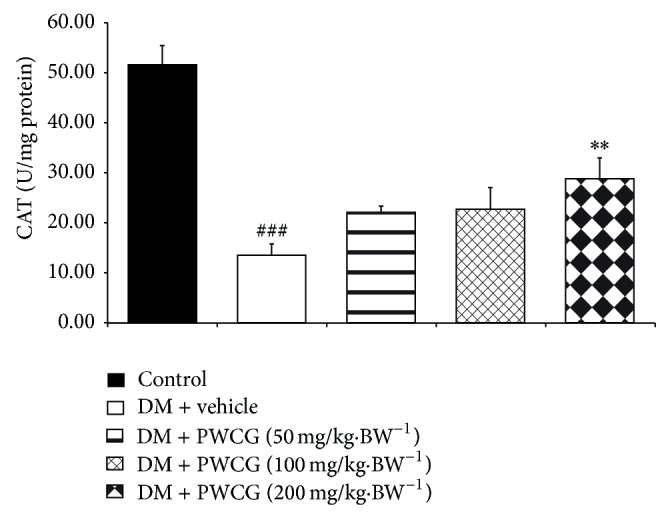
The activity of catalase (CAT) (U/mg protein) in lens of control and diabetic rats (DM) which received either vehicle or the combination extract of purple waxy corn and ginger (PWCG) at doses of 50, 100, and 200 mg·kg^−1^ BW for 10 weeks. (*N* = 8/group) ^###^ *P* value < 0.001, compared with control, and ^**^ *P* value < 0.01, compared with diabetic rats which received vehicle (DM + vehicle).

**Figure 12:**
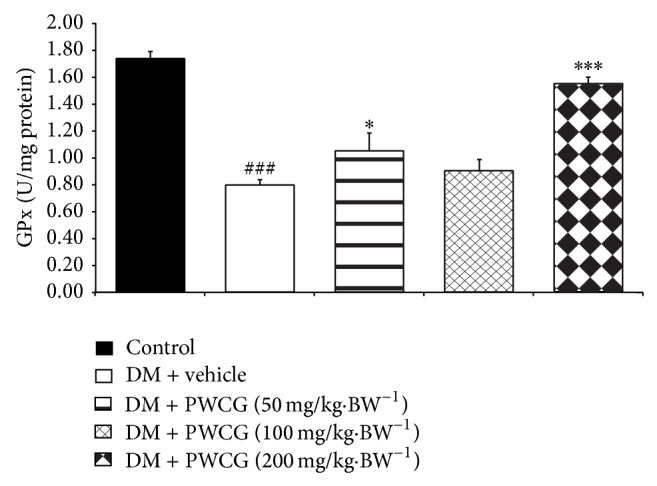
The activity of glutathione peroxidase (GPx) (U/mg protein) in lens of control and diabetic rats (DM) which received either vehicle or the combination extract of purple waxy corn and ginger (PWCG) at doses of 50, 100, and 200 mg·kg^−1^ BW for 10 weeks (*N* = 8/group). ^###^ *P* value < 0.001, compared with control, and  ^*^, ^***^ *P* value < 0.05, 0.001 respectively, compared with diabetic rats which received vehicle (DM + vehicle).

Based on the crucial role of oxidative stress on cataract and retinopathy, the effects of PWCG on oxidative stress markers including level of MDA and the activities of SOD, CAT, and GPx in lens were carried out. The results were shown in Figures 9, 10, 11, and 12. It was found that diabetic rats which received vehicle significantly increased MDA level (*P* value < 0.001, compared with control) but decreased SOD, CAT and GPx activities (*P* value < 0.001 all compared with control) in lens. PWCG at doses of 50, 100, and 200 mg·kg^−1^ BW significantly mitigated the elevation of MDA level induced by diabetes mellitus (*P* value < 0.001 all, compared with diabetic rats which received vehicle). Low dose of PWCG also mitigated the reduction of GPx activity in lens of diabetic rats (*P* value < 0.05, compared with diabetic rats which received vehicle) whereas high dose of PWCG mitigated the reduction of both CAT and GPx activities of lens of diabetic rats (*P* value < 0.01 and 0.001 resp., compared with diabetic rats which received vehicle). However, no significant changes of scavenger enzymes activities were observed in the diabetic rats which received medium dose of PWCG.

### 3.5. Effect of PWCG on Aldose Reductase Activity

**Figure 13:**
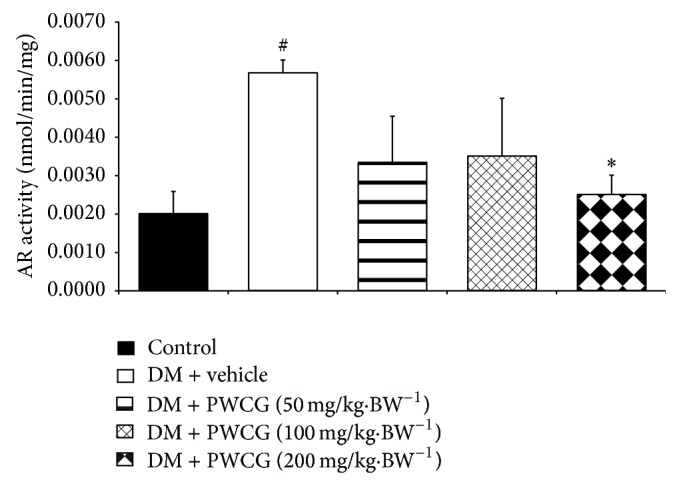
The activity of aldose reductase (AR) (nmol/min/mg) in lens of control and diabetic rats (DM) which received either vehicle or the combination extract of purple waxy corn and ginger (PWCG) at doses of 50, 100, and 200 mg·kg^−1^ BW for 10 weeks (*N* = 8/group). ^#^ *P* value < 0.05, compared with control, and ^*^ *P* value < 0.05, compared with diabetic rats which received vehicle (DM + vehicle).

In addition to the oxidative stress, the polyol pathway also plays the crucial role on the cataractogenesis. Therefore, the effect of PWCG on aldose reductase enzyme activity in lens was also investigated and data were shown in Figure 13. It was demonstrated that aldose reductase activity in lens of diabetic rats was increased (*P* value < 0.05, compared with control). PWCG at dose of 200 mg·kg^−1^ BW could significantly decrease the elevation of aldose reductase induced by diabetes mellitus in rat lens. No other significant changes were observed.

## 4. Discussion

Diabetic cataract and retinopathy are the important causes of blindness so the prevention of these conditions is very much important. Our results clearly demonstrated that PWCG significantly mitigated the reduction of ganglia in outer nuclear layer (ONL), OUL thickness, and total retina thickness. In addition, PWCG also showed anticataract effect. Together with the changes mentioned earlier, the decreased oxidative stress and aldose reductase activity in rat lens were also observed.

It has been reported that the neurodegeneration of ganglion cells plays the crucial role on the reduction of OUL and total retina thickness [21]. The degeneration of retinal ganglion cells is under the influence of many factors such as inflammation, oxidative stress, or exposure to advanced glycation end products [22] and polyol pathway [23]. Our data also demonstrates the increased oxidative stress markers such as MDA level and the enhanced aldose reductase activity in lens of diabetic rats. These changes are in agreement with the previous study [22, 23]. Therefore, we suggested that the effect of PWCG to attenuate retinopathy induced by diabetes mellitus might be partly related to the decreased oxidative stress and aldose reductase activity in rat lens. It was found that the enhanced of GPx activity in rat lens might be responsible in part for the reduction of oxidative stress in rat lens of diabetic rats especially at low and high doses of PWCG. In addition to the increased GPx activity, the high dose of PWCG also enhanced CAT in rat lens. Therefore, the elevation of both CAT and GPx might be responsible for the decreased oxidative stress which in turn enhanced the survival of ganglion cells in OUL leading to the increased total retina thickness. However, the decreased oxidative stress in rat lens induced by medium dose of PWCG might be associated with other mechanism in addition to the elevation of the scavenger enzymes activities. Since the excess oxidative stress occurs as the result of imbalance between oxidative stress formation and oxidative stress buffering capacities, we suggested that the decreased oxidative stress formation might play a role on the reduction of oxidative stress induced by the PWCG especially at the medium concentration.

Oxidative stress and aldose reductase also play the crucial role on the cataractogenesis in diabetic rats [24, 25]. In addition, it has been reported that antioxidant and aldose reductase inhibitory effects can improve cataract [26] Thus, the anticataractogenis of PWCG might also be associated with both effects mentioned earlier. Aldose reductase also plays a role on the pathophysiology of diabetic retinopathy [27]. Based on the basis of this information, we do suggest that the effect of PWCG to improve retinopathy especially at high dose should also relate with the aldose reductase inhibitory effect.

Previous study has demonstrated that flavonoids can protect against oxidative stress and increase the survival of ganglion cells [28] and prevent cataract [29] in diabetic rats. Recent study has demonstrated that anthocyanins and anthocyanins-rich substance have the potential to protect against retinopathy [30] and diabetic cataract [26]. In addition, ginger and its constituents also show the potential to protect against diabetic retinopathy [31]. Therefore, the anticataractogenesis and antiretinopathy of PWCG may be due to the flavonoids such as anthocyanins in PWCG. Other types of flavonoid may also play a role. However, further researches are required to confirm the possible active ingredient.

In this study, the slit lamp examination clearly provided the changes of lens characteristic. However, no pupil was dilated during the grading of severity as that was performed during the slit lamp examination in human. This may possibly influence the precision of grading scores which reflect the real severity of cataract. To obtain the precise severity of cataract, the slit lamp examination should be performed while pupil was dilated. However, all rats were assessed under the condition without the pupil dilator so no confounding error about the beneficial effect of PWCG on cataract was achieved.

The lenticular opacification in control rats was also observed during the 6–10 weeks of study period. Based on the previous study that stress hormone or glucocorticoids can also induce cataract [32, 33], we do suggest that the development of cataract in control group may possibly occur as the exposure to stress condition leading to the elevation of glucocorticoids hormone resulting in the cataractogenesis. Unfortunately, the level of corticosterone has not been determined in this study. Therefore, the precise understanding mechanism of this change requires further investigation. In addition, spontaneous age-related cataract can also occur in laboratory rodents. The prevalence is varied depending on species. Even young adolescent rodents can be found to have cataract, approximately 20% [34]. Therefore, the cataract observed in normal rats might occur either via stress or via spontaneous cataractogenesis of the laboratory rodents mentioned earlier.

## 5. Conclusion

The combination extract of purple waxy corn seeds and ginger rhizome (PWCG) can protect against diabetic cataract and diabetic retinopathy. The possible underlying mechanism may occur partly via the decreased oxidative stress and the suppression of aldose reductase activity in lens. The possible active ingredient may be associated with flavonoids such as anthocyanin and other flavonoids. Further researches are still essential to understand the precise underlying mechanism and possible active ingredient.

## Figures and Tables

**Table 1:** Opacity index of the lens of normal and diabetic rats (DM) which received either vehicle or PWCG at doses of 50, 100, and 200 mg·kg^−1^ BW for 10 weeks (*N* = 8/group). ^##^, ^###^ *P* value < 0.01 and 0.001, respectively, compared with control group. ^*^, ^**^, ^***^ *P* value < 0.05, 0.01, and 0.001 respectively, compared with diabetic rats which received vehicle (DM + vehicle).

Time duration	Opacity index of the lens (mean ± SEM)				
	Control	DM + vehicle	DM + PWCG (50 mg/kg·BW^−1^)	DM + PWCG (100 mg/kg·BW^−1^)	DM + PWCG (200 mg/kg·BW^−1^)
Baseline	0.00 ± 0.00	0.00 ± 0.00	0.00 ± 0.00	0.00 ± 0.00	0.00 ± 0.00
Week 1	0.00 ± 0.00	0.00 ± 0.00	0.00 ± 0.00	0.00 ± 0.00	0.00 ± 0.00
Week 2	0.00 ± 0.00	0.06 ± 0.06	0.00 ± 0.00	0.00 ± 0.00	0.00 ± 0.00
Week 3	0.00 ± 0.00	0.50 ± 0.16^##^	0.14 ± 0.10^*^	0.50 ± 0.13	0.22 ± 0.10
Week 4	0.00 ± 0.00	1.00 ± 0.20^###^	0.79 ± 0.24	1.00 ± 0.18	0.72 ± 0.23
Week 5	0.00 ± 0.00	1.81 ± 0.31^###^	1.00 ± 0.21^*^	1.25 ± 0.23	1.00 ± 0.28^*^
Week 6	0.08 ± 0.08	2.25 ± 0.32^###^	1.50 ± 0.29	1.50 ± 0.22	1.11 ± 0.31^**^
Week 7	0.17 ± 0.11	2.50 ± 0.34^###^	1.71 ± 0.29	1.88 ± 0.29	1.44 ± 0.41^*^
Week 8	0.25 ± 0.13	3.06 ± 0.23^###^	2.00 ± 0.30^*^	2.19 ± 0.25^*^	1.67 ± 0.40^***^
Week 9	0.50 ± 0.15	3.81 ± 0.10^###^	2.29 ± 0.35^***^	2.38 ± 0.26^***^	1.78 ± 0.39^***^
Week 10	0.50 ± 0.15	4.00 ± 0.00^###^	2.21 ± 0.37^***^	2.63 ± 0.26^***^	1.89 ± 0.40^***^
